# Sarcopenia and cognitive impairment: a multidimensional study of clinical associations, shared genetics, and causal links

**DOI:** 10.3389/fnagi.2025.1708170

**Published:** 2025-12-01

**Authors:** Kemeng Zhang, Sui Huang, Mengchen Liu, Yi Zhang, Wenhan Li, Bijin Luo, Ping He

**Affiliations:** Department of Geriatrics, Union Hospital, Tongji Medical College, Huazhong University of Science and Technology, Wuhan, Hubei, China

**Keywords:** sarcopenia, cognitive impairment, genetic correlation, pleiotropic analysis, Mendelian randomization

## Abstract

**Objective:**

To comprehensively investigate the relationship between sarcopenia and cognitive impairment by examining their clinical associations, shared genetic architecture, and potential causal links, using a multidimensional approach.

**Methods:**

To assess the sarcopenia and cognitive impairment risk, multivariable-adjusted logistic regression was conducted on Wuhan Junshan Community data. Utilizing large-scale GWAS summary statistics, we identified potential genetic overlaps between sarcopenia and cognitive impairment. Cross-trait pleiotropic analyses were conducted to uncover shared genetic loci and pleiotropic genes between these conditions. Comprehensive functional annotation and tissue-specific expression analyses were then performed to characterize the biological roles of these shared genetic factors. Finally, we employed Mendelian randomization (MR) approaches to examine potential causal relationships between sarcopenia and cognitive impairment.

**Results:**

In this study, we recruited 575 participants for this observational study. Multivariable-adjusted logistic regression revealed a significant positive association between sarcopenia and cognitive impairment risk (OR = 3.26, 95% CI: 1.65 to 6.42). Genomic analysis revealed that there was a significant genetic correlation between sarcopenia and cognitive impairment, and 19 pairs of significantly correlated trait combinations were identified. Pleiotropic analysis revealed 79 risk loci and 428 pleiotropic genes such as FoxO3 and SLC39A8, which were enriched in neurodegenerative pathway and FoxO signaling pathway. MR analysis showed that appendicular lean mass and usual walking pace had potential causal protective effects on cognitive function, while low hand grip strength had the opposite effect.

**Conclusion:**

This study provides evidence for both clinical and genetic links between sarcopenia and cognitive impairment, uncovering their potential biological mechanisms.

## Introduction

Sarcopenia (characterized by progressive loss of muscle mass and function) and cognitive impairment demonstrate significant comorbidity in the elderly population, forming a vicious cycle that not only accelerates physical functional decline but also substantially impairs quality of life (Yuan and Larsson, 2023; Cipolli et al., 2023). Epidemiological studies reveal that sarcopenic patients have a 20.5% increased relative risk of developing mild cognitive impairment (MCI) compared to the general elderly population, with a significantly higher probability of progression to Alzheimer’s disease (AD) (Yang et al., 2023; Beeri et al., 2021). Notably, this association exhibits distinct bidirectional characteristics: patients with MCI experience an annual muscle mass loss rate 1.5 times higher than age-matched healthy controls, accompanied by more pronounced declines in muscle strength (Deng et al., 2021).

Against the backdrop of global population aging, the comorbidity of sarcopenia and cognitive impairment has emerged as a critical public health challenge requiring urgent attention. Current epidemiological data reveal a 14.2% prevalence of comorbid sarcopenia and cognitive impairment in adults aged 65 years and older (Zengarini et al., 2019). More alarmingly, the synergistic effects of these conditions demonstrate a remarkable multiplicative impact: clinical research data show that compared to patients with only cognitive impairment or sarcopenia, those with comorbid sarcopenia and cognitive impairment exhibit a greater mortality risk (OR = 2.12, 95% CI: 1.05–4.13) (Zengarini et al., 2019). These findings underscore the substantial burden this comorbidity places on healthcare systems. Although the association between sarcopenia and cognitive impairment has been widely explored, there is still insufficient high-quality evidence for the specific population of elderly people aged 60 and above in real community environments, and further supplementation is urgently needed.

The close association between sarcopenia and cognitive impairment may stem from shared pathophysiological mechanisms (Sui et al., 2020). The upregulation of chronic inflammatory mediators (IL-6, TNF-α) coordinately induces proteolytic muscle breakdown and hippocampal neurotoxicity (Sharma and Dabur, 2020; Swardfager et al., 2010). Insulin resistance exerts dual detrimental effects by impairing cerebral glucose metabolism and suppressing muscle protein synthesis (Umegaki et al., 2017). Mitochondrial dysfunction leads to oxidative stress, manifested by decreased NAD + levels and reduced respiratory chain complex activity, ultimately contributing to both muscle atrophy and neuronal damage (Arosio et al., 2023). Gut microbiota dysbiosis influences systemic function through the gut-brain-muscle axis, altering barrier permeability (Ticinesi et al., 2019). The decline in muscle-derived irisin and brain-derived neurotrophic factor (BDNF) disrupts the critical cross-talk between muscle and nervous system, leading to impaired neuromuscular signaling pathways (Lee et al., 2023). Lifestyle factors are also likely to mediate the association between sarcopenia and cognitive impairment (Sui et al., 2020). Specifically, sedentary habits and poor nutrition could enhance chronic low-grade inflammation, which have been shown to promote muscle wasting and exacerbate neurodegeneration (Mo et al., 2023; Diniz et al., 2024). These pathogenic mechanisms interact synergistically, forming a vicious cycle that accelerates the progressive decline of both muscular and cognitive functions. Notably, single-disease interventions (cognitive training or resistance exercise) may demonstrate limited effectiveness in comorbid conditions (Casas-Herrero et al., 2022), highlighting the necessity to investigate the deeper shared pathological pathways.

**Figure 1:**
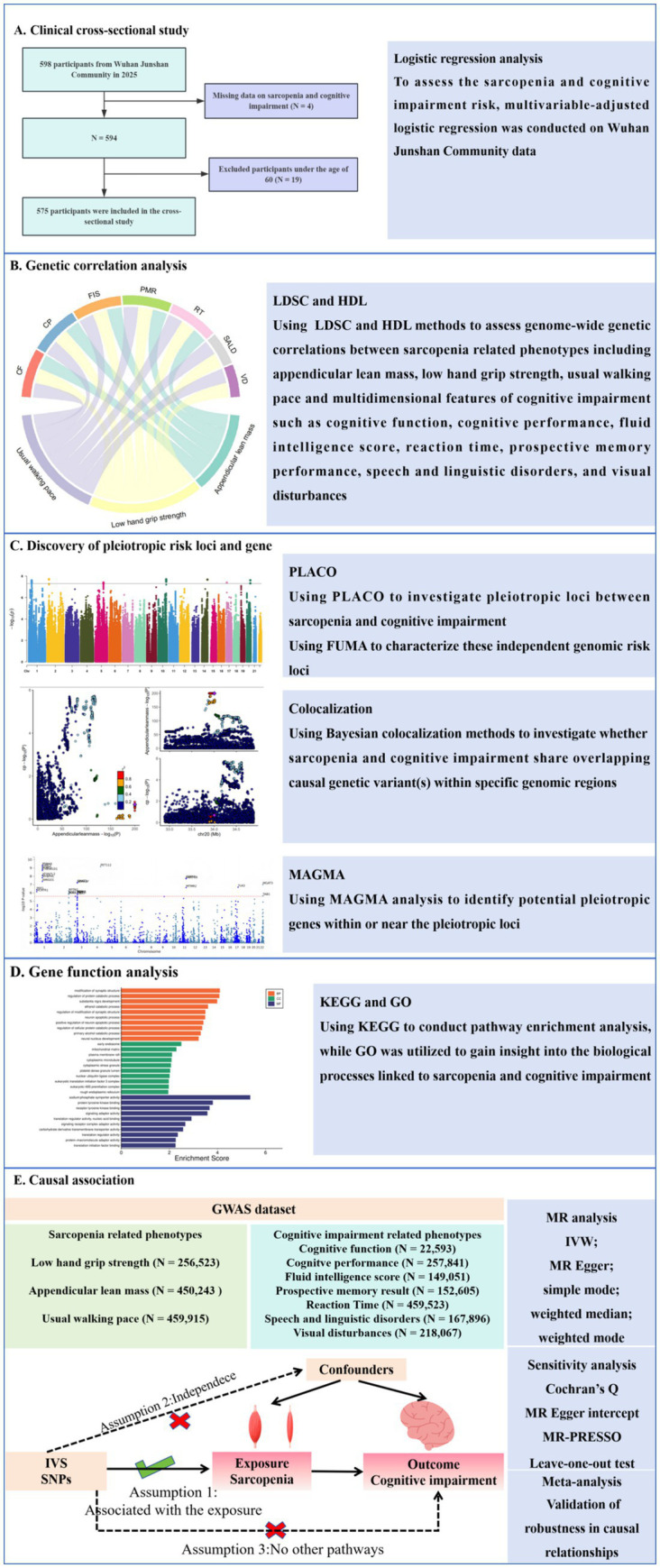
Overall study flowchart.

To establish clinical context for sarcopenia-cognitive interactions, we first conducted an observational study in Wuhan Junshan Community (2025) that systematically evaluated the relationship between sarcopenia and cognitive impairment. Building on these clinical findings, we then investigated the shared genetic architecture between sarcopenia and cognitive impairment. Although genome-wide association studies (GWAS) have independently identified sarcopenia-associated genes (MYH1 and MYH2, which encode myosin) (Kedlian et al., 2024) and cognitive impairment risk loci (APOE and CLU, neurodegeneration-related genes) (Bellenguez et al., 2022), the shared genetic architecture between these two disorders remains to be elucidated, with several critical research gaps persisting: (1) whether cross-phenotype pleiotropic genetic variants exist; (2) how these shared genetic factors mediate the coordinated decline of the neuromuscular system through specific molecular pathways; and (3) whether their genetic correlations exhibit tissue-specific expression patterns. Elucidating these mechanisms will provide molecular targets for developing precision intervention strategies targeting the “muscle-brain axis.” The study flowchart is shown in Figure 1.

## Materials and methods

### Clinical study

The data were obtained from Wuhan Junshan Community in 2025. This study has been approved by the Ethics Committee of Tongji Medical College Affiliated Union Hospital of Huazhong University of Science and Technology (0256–01). This study excluded individuals under the age of 60 and those lacking assessment of sarcopenia and cognitive impairment, and ultimately included 575 participants. Baseline characteristics in study population were summarized based on cognitive impairment status. Data were presented as means ± standard deviation (SD) or median and interquartile range (IQR) for continuous variables and percentages (%) for categorical variables. The *t*-test or Mann–Whitney U test and the chi-squared test or Fisher’s exact test were used for continuous and categorical variables, respectively, to analyze differences in baseline characteristics between non-cognitive impairment and cognitive impairment individuals. Multivariable-adjusted logistic regression analysis was used to calculate the odds ratio (OR) and 95% confidence interval (95% CI) for cognitive impairment to sarcopenia after adjusting for confounding factors.

### Source of GWAS data

The open GWAS database established by the MRC Integrated Epidemiology Unit (IEU) provided the majority of the summary-level data used in this investigation (Hemani et al., 2018). Data for sarcopenia related phenotypes including appendicular lean mass (ALM), low hand grip strength (LHGS), usual walking pace (UWP) and multidimensional features of cognitive impairment such as cognitive function (CF), cognitive performance (CP), fluid intelligence score (FIS), reaction time (RT), prospective memory result (PMR), speech and linguistic disorders (SALD), and visual disturbances (VD) were derived from European ancestry (Supplementary Table 1 contains the complete phenotypic descriptions), with no sample overlap between the datasets of the pairwise traits.

### Genetic correlation analysis

To investigate shared genetic architecture, we employed linkage disequilibrium score regression (LDSC) (Bulik-Sullivan et al., 2015) and high-definition likelihood (HDL) (Ning et al., 2020) methods to assess genome-wide genetic correlations between sarcopenia related phenotypes and multidimensional features of cognitive impairment. LDSC calculates SNP-specific linkage disequilibrium (LD) scores by summing squared correlations (r^2^) with neighboring variants within a 1-centimorgan window using reference panels (1,000 Genomes Project) (Abecasis et al., 2012), then estimates heritability through χ^2^ statistic regression while controlling for confounding biases. HDL enhances this approach by incorporating haplotype-level information from high-resolution references (UK Biobank) (Bycroft et al., 2018) within a likelihood framework, improving precision (30–50% efficiency gains) through refined modeling of regional LD complexity. Both methods were applied to quality-controlled HapMap3 SNPs (Duan et al., 2008), with HDL providing complementary validation of LDSC results in examining shared genetic architecture between these traits. To account for multiple hypothesis testing, we implemented Benjamini-Hochberg false discovery rate (FDR) (Robertson et al., 2023) control, considering results with q-values below 0.05 as statistically significant.

### Discovery of pleiotropic risk loci

Pleiotropic analysis under composite null hypothesis (PLACO) (Ray and Chatterjee, 2020) was employed to investigate shared genetic loci between sarcopenia-related traits and cognitive impairment measures using GWAS association statistics. Genome-wide significant pleiotropic variants were identified as single nucleotide variants (SNVs) meeting the rigorous PLACO significance criterion of *p* < 5 × 10^−8^. For comprehensive functional characterization of these independent genomic risk loci, we conducted subsequent bioinformatic analyses utilizing the Functional Mapping and Annotation (FUMA) platform (Watanabe et al., 2017).

### Colocalization analysis

We performed genetic colocalization analysis to investigate whether sarcopenia and cognitive impairment share overlapping causal genetic variant(s) within specific genomic regions, thereby strengthening evidence for their biological linkage. Using Bayesian colocalization methods (Giambartolomei et al., 2014), we analyzed GWAS summary statistics for sarcopenia-related traits and multidimensional features of cognitive impairment. The analysis evaluated five competing hypotheses: the null hypothesis (H0) of no association with either trait; H1 (association with sarcopenia only); H2 (association with cognitive impairment only); H3 (independent associations for both traits); and H4 (shared causal variant). Loci demonstrating colocalization were identified when the posterior probability for H4 (PP4) exceeded our threshold of 0.75, indicating strong evidence that both traits are driven by the same underlying genetic variant(s) in these genomic regions.

### Identification of pleiotropic gene and additional tissue specific analysis

To identify potential pleiotropic genes within or near the pleiotropic loci, we performed gene-level multi-marker analysis of genomic annotation (MAGMA) (De Leeuw et al., 2015) using our PLACO results, which provided biological insights into the pleiotropic SNPs. The MAGMA analysis *p*-values were adjusted for multiple comparisons using false discovery rate (FDR) correction, accounting for the number of genes tested per trait, with an FDR q-value threshold of < 0.05 defining statistical significance. The MAGMA analysis was conducted within the FUMA framework (Watanabe et al., 2017).

Pathway enrichment analysis was performed using the KEGG Orthology-Based Annotation System (version 3.0) (Bu et al., 2021), a comprehensive resource from the Kyoto Encyclopedia of Genes and Genomes. To elucidate the biological mechanisms underlying sarcopenia and cognitive impairment, we employed Gene Ontology (GO) enrichment analysis.

### MR analysis and meta-analysis

MR represents an established epidemiological approach that leverages genetic variants (single nucleotide polymorphisms, SNPs) as instrumental variables to infer causal relationships between exposures and outcomes (Liu et al., 2022). During the clumping procedure, we applied stringent thresholds for linkage disequilibrium (r^2^ < 0.001) and physical distance (>10 Mb) using the European population reference data from the 1,000 Genomes Project for LD calculation (Clarke et al., 2012). In our Mendelian randomization analysis, sarcopenia served as the exposure variable, with a stringent genome-wide significance threshold of *p* < 5 × 10^−8^ applied for instrumental variable selection (Chen et al., 2021).

To ensure the robustness of our MR causal estimates, we conducted comprehensive sensitivity analyses employing multiple methodological approaches (Burgess et al., 2017). The effect estimates were meta-analyzed using a DerSimonian-Laird random-effects model, with between-study heterogeneity evaluated through Cochran’s Q statistic (Bowden et al., 2018).

In MR studies, the inverse-variance weighted (IVW) estimates may be susceptible to bias from horizontal pleiotropy. To address this potential limitation, we implemented multiple pleiotropy-robust sensitivity analyses, including MR-Egger regression to assess and account for directional pleiotropic effects among the instrumental variables (Bowden et al., 2015). Statistical evidence of directional pleiotropy among the instrumental variables (IVs) was considered significant when the MR-Egger intercept term yielded a *p*-value < 0.05. Conversely, the absence of significant horizontal pleiotropy was concluded when the intercept p-value exceeded 0.05, indicating that the selected IVs satisfied the key MR assumption of no unbalanced pleiotropy. Prior to conducting each Mendelian randomization analysis, we implemented the MR-Pleiotropy RESidual Sum and Outlier (MR-PRESSO) method to identify and remove potential outlier variants that could distort causal estimates (Yeung et al., 2019). To verify robustness, we conducted leave-one-out sensitivity analyses.

To enhance statistical power, we performed a meta-analysis of multiple MR results. All analyses were performed using R software (version 4.3.0).

## Results

### Characteristics of participants

A total of 575 older adults were included in the cross-sectional analyses. The characteristics of the participants were shown in Table 1 according to cognitive impairment status. The prevalence of cognitive impairment was 29.2% among old people. Compared with those without cognitive impairment, individuals with cognitive impairment were more likely to be older (age: 75.0 vs. 71.0 years), slimmer (BMI: 23.35 vs. 24.30 kg/m^2^), higher risk of malnutrition (MNA-SF score: 11.00 vs. 12.00), lower levels of daily activities (ADL score: 95.00 vs. 100.0) and with educational background of primary school or below (76.19% vs. 63.46%). With regard to comorbidity, sarcopenic individuals were more likely to suffer from cerebrovascular disease (33.33% vs. 24.63%), dysaudia(32.14% vs. 23.25%), visualdisturbance (42.51% vs. 30.77%). The marital status of patients with cognitive impairment is mostly divorced or other (25.00% vs. 16.95%). Besides, patients with cognitive impairment have lower muscle mass (ASMI:6.77 vs. 7.15 kg/m^2^), slower walking speed (0.67 vs. 0.80 m/s), and longer time of 5-time chair stand test (13.00 vs. 12.00 s). The prevalence of sarcopenia (35.12% vs. 16.19%) is higher in cognitive impairment.

**Table 1:** Baseline characteristics of participants.

Variable	Total (*n* = 575)	Non-cognitive impairment (*n* = 407)	Cognitive impairment (*n* = 168)	*p* value
Age (years)	72.00 (69.00, 76.00)	71.00 (68.00, 76.00)	75.00 (71.00, 78.00)	< 0.001
Gender, *n* (%)				0.08
Male	220 (38.26)	165 (40.54)	55 (32.74)	
Female	355 (61.74)	242 (59.46)	113 (67.26)	
BMI (kg/m^2^)	24.00 (21.95, 26.35)	24.30 (22.30, 26.50)	23.35 (21.40, 25.92)	0.021
Smoking, *n* (%)	84 (14.74)	63 (15.63)	21 (12.57)	0.349
Drinking, *n* (%)	119 (20.88)	90 (22.33)	29 (17.37)	0.184
Education level, *n* (%)				0.009
Primary school or below	385 (67.19)	257 (63.46)	128 (76.19)	
Middle school	150 (26.18)	116 (28.64)	34 (20.24)	
High school or above	38 (6.63)	32 (7.90)	6 (3.57)	
Marital status, *n* (%)				0.026
Married	464 (80.70)	338 (83.05)	126 (75.00)	
Divorced and other	111 (19.30)	69 (16.95)	42 (25.00)	
MNA-SF (scores)	12.00 (11.00, 12.00)	12.00 (11.00, 12.00)	11.00 (10.00, 12.00)	0.008
ADL (scores)	100.0 (95.00, 100.0)	100.0 (95.00, 100.0)	95.00 (85.00, 100.0)	< 0.001
Hypertension, *n* (%)	407 (70.78)	287 (70.52)	120 (71.43)	0.827
Diabetes, *n* (%)	98 (17.04)	70 (17.20)	28 (16.67)	0.877
Coronary heart disease, *n* (%)	129 (22.47)	84 (20.69)	45 (26.79)	0.111
Cerebrovascular disease, *n* (%)	156 (27.18)	100 (24.63)	56 (33.33)	0.033
Hyperlipidemia, *n* (%)	173 (30.09)	126 (30.96)	47 (27.98)	0.478
Dysaudia, *n* (%)	147 (25.88)	93 (23.25)	54 (32.14)	0.027
Visualdisturbances, *n* (%)	195 (34.21)	124 (30.77)	71 (42.51)	0.007
Waist (cm)	88.00 (80.00, 94.00)	88.00 (80.50, 95.00)	86.00 (80.00, 92.50)	0.113
Walk speed (m/s)	0.80 (0.67, 0.80)	0.80 (0.67, 0.80)	0.67 (0.57, 0.80)	< 0.001
5-time chair stand test (s)	13.00 (11.00, 15.00)	12.00 (11.00, 14.00)	13.00 (12.00, 16.00)	< 0.001
ASMI (kg/m^2^)	7.01 (5.92, 8.05)	7.15 (6.03, 8.18)	6.77 (5.58, 7.73)	0.006
Low hand grip strength, *n* (%)	212 (36.87)	106 (26.04)	106 (63.10)	< 0.001
Sarcopenia, *n* (%)	128 (22.26)	69 (16.95)	59 (35.12)	< 0.001

BMI, body mass index; MNA-SF, short-form mini-nutritional assessment; ADL, activities of daily living; ASMI, appendicular skeletal muscle mass index.

### Clinical association between sarcopenia and cognitive impairment

The results of a multivariate regression analysis on the relationship between sarcopenia and cognitive impairment are shown in Table 2. The OR (95% CI) for cognitive impairment in sarcopenia old adults was 2.65 (95% CI: 1.76 to 3.99). The sarcopenia was significantly associated with cognitive impairment after adjusting for age, gender, BMI, education level, marital status, MNA-SF scores, ADL scores, cerebrovascular disease, dysaudia, visualdisturbances (OR = 3.26, 95% CI: 1.65 to 6.42, *p* < 0.001). We found similar results in the regression analysis of the relationship between low grip strength, low muscle mass and cognitive impairment (*p* < 0.05).

**Table 2:** Associations of the sarcopenia with cognitive impairment.

Variable	Model 1	*p* value	Model 2	*p* value	Model 3	*p* value	Model 4	*p* value
	OR (95%CI)		OR (95%CI)		OR (95%CI)		OR (95%CI)	
Sarcopenia								
No	1.00 (Reference)		1.00 (Reference)		1.00 (Reference)		1.00 (Reference)	
Yes	2.65 (1.76 ~ 3.99)	<0.001	2.82 (1.59 ~ 5.03)	<0.001	3.18 (1.63 ~ 6.22)	<0.001	3.26 (1.65 ~ 6.42)	< 0.001
Low muscle mass								
No	1.00 (Reference)		1.00 (Reference)		1.00 (Reference)		1.00 (Reference)	
Yes	2.01 (1.36 ~ 2.99)	<0.001	1.85 (1.03 ~ 3.32)	0.040	2.14 (1.05 ~ 4.34)	0.035	2.20 (1.08 ~ 4.50)	0.031
Low hand grip strength								
No	1.00 (Reference)		1.00 (Reference)		1.00 (Reference)		1.00 (Reference)	
Yes	4.85 (3.31 ~ 7.13)	<0.001	4.19 (2.81 ~ 6.26)	<0.001	4.00 (2.65 ~ 6.03)	<0.001	4.03 (2.65 ~ 6.12)	< 0.001
Low walk speed								
No	1.00 (Reference)		1.00 (Reference)		1.00 (Reference)		1.00 (Reference)	
Yes	2.37 (1.33 ~ 4.21)	0.003	1.86 (1.01 ~ 3.46)	0.049	1.76 (0.92 ~ 3.35)	0.085	1.70 (0.89 ~ 3.26)	0.107

Model 1 not adjusted; Model 2 adjusted for age, BMI, gender; Model 3 adjusted for the variables in Model 2 and educational level, marital status, MNA-SF scores, ADL scores; Model 4 adjusted for the variables in Model 3 and cerebrovascular disease, dysaudia, visualdisturbances.

### Genetic correlation between sarcopenia and cognitive impairment

Using LDSC analysis, we identified 19 trait pairs showing significant genetic correlations between sarcopenia and cognitive impairment phenotypes (FDR-adjusted *p* < 0.05). These findings demonstrate high consistency with results obtained from the HDL approach, further validating the existence of significant shared genetic architecture between these two phenotypes. Both ALM and UWP exhibited significant positive genetic correlations with cognitive function, cognitive performance, and fluid intelligence scores. Significant negative genetic correlations were observed between ALM/UWP and both reaction time and prospective memory performance. UWP additionally showed significant negative genetic associations with speech and linguistic disorders as well as visual disturbances. In contrast, LHGS displayed an inverse pattern, showing negative genetic correlations with cognitive function, cognitive performance and fluid intelligence scores, but positive correlations with reaction time, prospective memory performance, speech and linguistic disorders, and visual disturbances (Table 3; Supplementary Figure 1).

**Table 3:** Genetic correlation analysis between sarcopenia and cognitive impairment.

Trait pairs	LDSC		HDL	
	r_g_ (SE)	FDR *q*-value	r_g_ (SE)	FDR *q*-value
ALM-Cognitive function	0.202 (0.034)	7.04E-09	0.183 (0.033)	5.25E-08
ALM-Cognitive performance	0.150 (0.016)	1.56E-19	0.137 (0.011)	4.94E-33
ALM-Fluid intelligence score	0.153 (0.016)	1.60E-19	0.135 (0.011)	4.94E-33
ALM-Reaction time	−0.063 (0.018)	0.001	−0.062 (0.012)	1.41E-07
ALM-Prospective memory result	−0.071 (0.025)	0.008	−0.080 (0.013)	2.49E-09
ALM-Speech and linguistic disorders	−0.065 (0.069)	0.349	−0.480 (0.261)	0.077
ALM-Visualdisturbances	−0.066 (0.042)	0.137	−0.037 (0.041)	0.366
LHGS-Cognitive function	−0.135 (0.053)	0.012	−0.167 (0.053)	0.002
LHGS-Cognitive performance	−0.085 (0.028)	0.007	−0.106 (0.015)	1.78E-11
LHGS-Fluid intelligence score	−0.086 (0.029)	0.007	−0.118 (0.184)	3.22E-10
LHGS-Reaction time	0.164 (0.027)	1.35E-08	0.194 (0.022)	2.16E-19
LHGS-Prospective memory result	0.107 (0.042)	0.012	0.216 (0.024)	2.18E-18
LHGS-Speech and linguistic disorders	0.336 (0.117)	0.007	1.369 (0.527)	0.009
LHGS-Visualdisturbances	0.180 (0.063)	0.007	0.250 (0.090)	0.006
UWP-Cognitive function	0.373 (0.042)	7.40E-18	0.390 (0.045)	8.14E-18
UWP-Cognitive performance	0.325 (0.021)	4.10E-55	0.320 (0.013)	6.10E-131
UWP-Fluid intelligence score	0.328 (0.021)	2.50E-53	0.333 (0.013)	4.86E-143
UWP-Reaction time	−0.071 (0.022)	0.001	−0.089 (0.166)	1.48E-07
UWP-Prospective memory result	−0.215 (0.034)	4.25E-10	−0.235 (0.021)	1.94E-27
UWP-Speech and linguistic disorders	−0.350 (0.093)	2.40E-04	−1.78 (0.663)	0.007
UWP-Visualdisturbances	−0.151 (0.049)	0.002	−0.191 (0.066)	0.004

All *p*-values were adjusted for multiple comparisons using FDR correction and an FDR *q*-value < 0.05 was considered statistically significant. ALM, appendicular lean mass; LHGS, low hand grip strength; UWP, Usual walking pace; LDSC, linkage disequilibrium score regression; HDL, high-definition likelihood; r_g_, genetic correlation; SE, standard error; FDR, false discovery rate.

### Identification of pairwise pleiotropic loci and genes

**Figure 2:**
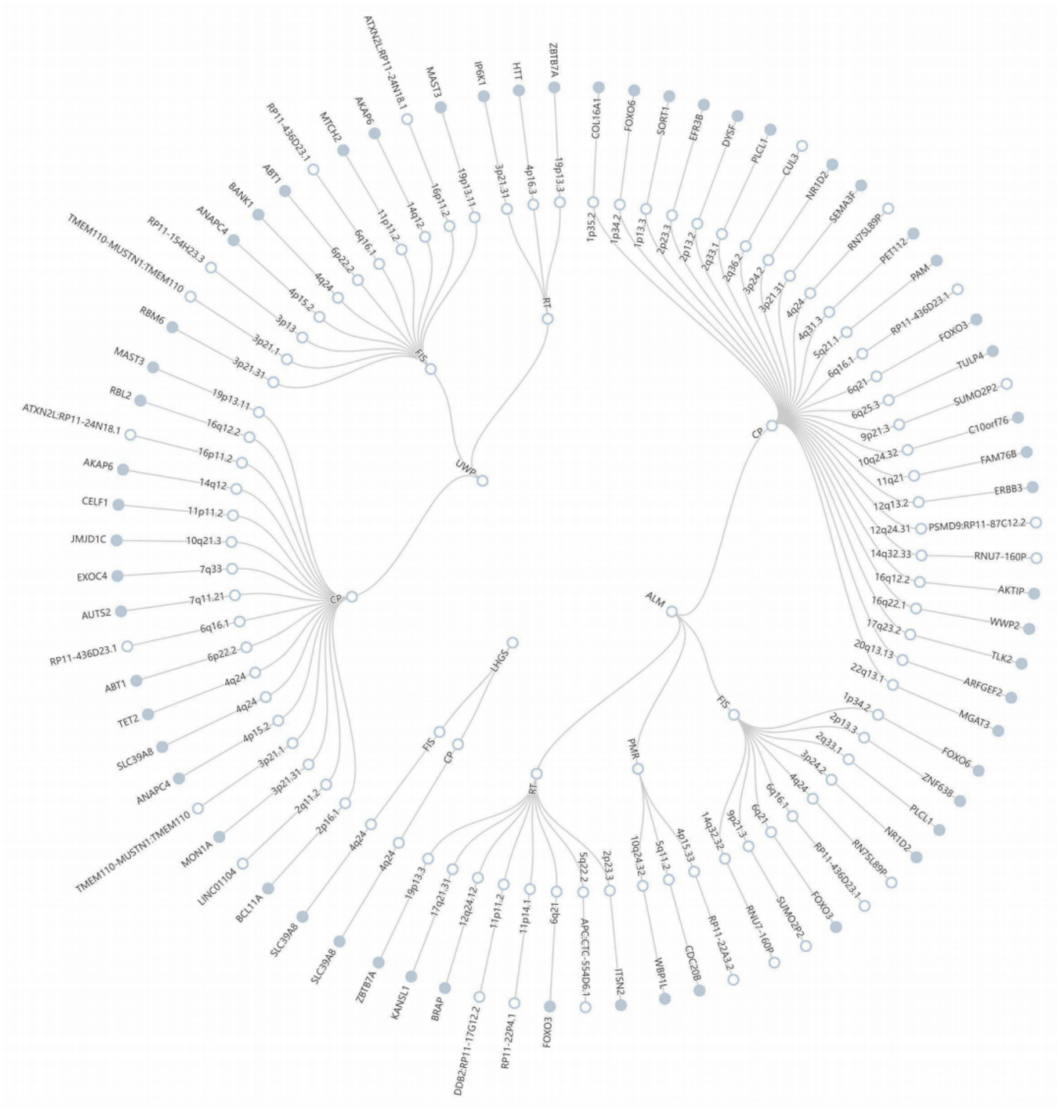
The circular diagram presents pleiotropic loci and genes identified by PLACO. Note: Shared genes identified by MAGMA analysis are highlighted in blue. ALM, appendicular lean mass; LHGS, low hand grip strength; UWP, Usual walking pace.

Building upon the evidence of shared genetic architecture between sarcopenia and cognitive impairment revealed through LDSC and HDL analyses, we employed the PLACO framework to detect genomic loci exhibiting pleiotropic effects across both conditions (Supplementary Figure 2). The QQ plots revealed close alignment between the observed and expected distributions of test statistics, providing no evidence for population stratification or other technical artifacts in our analyses (Supplementary Figure 3). The FUMA analysis identified 79 genome-wide significant pleiotropic risk loci (*P*_PLACO_ < 5 × 10^−8^), distributed across 49 distinct chromosomal regions, demonstrating shared genetic influences between the studied phenotypes (Figure 2; Supplementary Table 2). Colocalization analysis revealed no shared genetic causal loci between sarcopenia and cognitive impairment (Supplementary Table 3). Through MAGMA, we identified 45 recurrent pleiotropic genes showing consistent associations across multiple trait pairs. Notably, RP11-436D23.1 emerged as the most prominent pleiotropic gene, associated with four trait pairs, followed by FOXO3 and SLC39A8 (each linked to three pairs). Additional significant pleiotropic genes included FOXO6, PLCL1, NR1D2, ZBTB7A, ANAPC4, ABT1, AKAP6, and MAST3 (all associated with two trait pairs).

### Pleiotropic gene enrichment analysis

**Figure 3:**
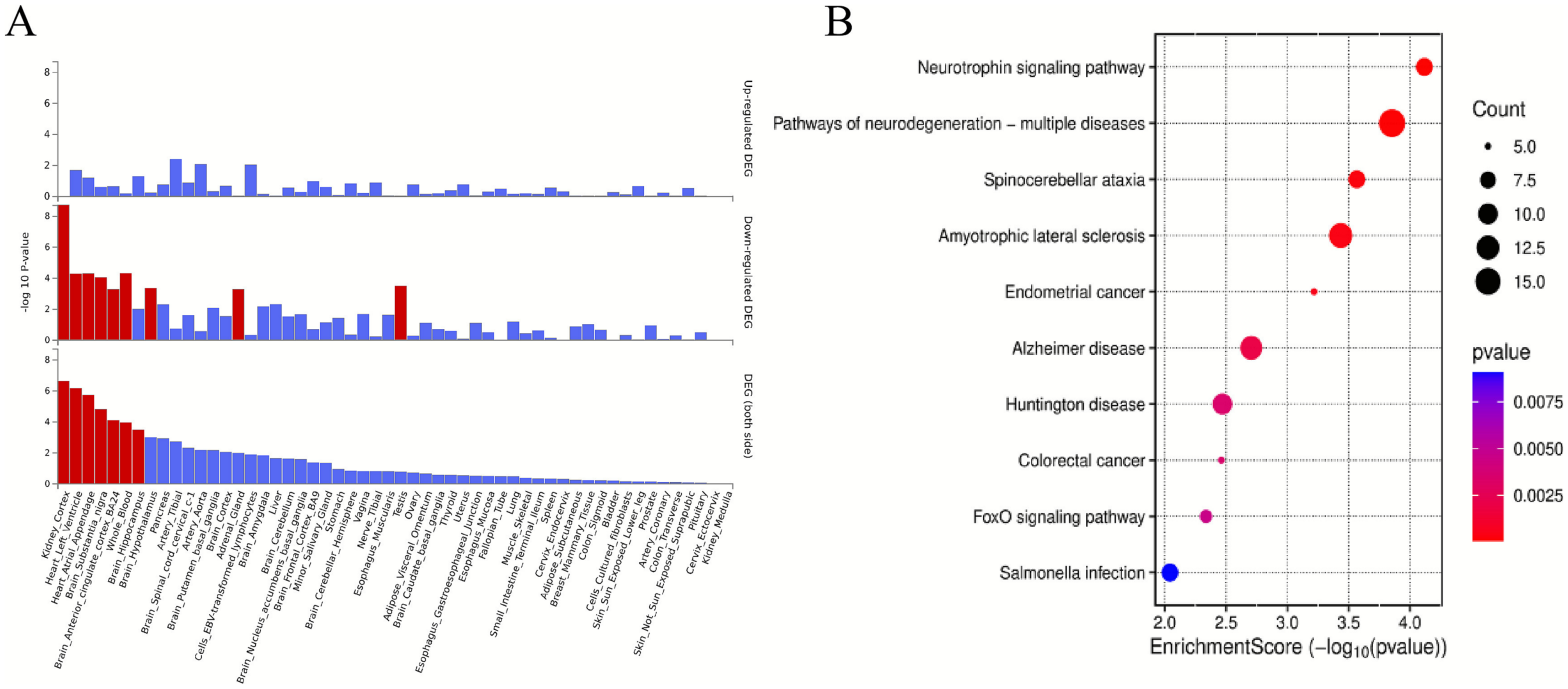
Enrichment analysis. **(A)** Enrichment analysis revealed differentially expressed pleiotropic genes in 54 GTEx tissues. Red bars denote significant enrichments (Bonferroni-adjusted). **(B)** Top 10 significant types of pathways in terms of the GO and KEGG enrichment analyses. 54 GTEx tissues, 54 Genotype-Tissue Expression tissues; DEGs, differentially expressed genes; GO, Gene Ontology, KEGG, Kyoto Encyclopedia of Genes and Genomes.

Integrative analysis using both PLACO and MAGMA approaches revealed a total of 428 statistically significant pleiotropic genes (FDR < 0.05) associated with both sarcopenia and cognitive impairment phenotypes (Supplementary Figure 4; Supplementary Table 4). The pleiotropic genes exhibited tissue-specific expression patterns across multiple organs, such as kidneys, heart, brain, and blood (Figure 3A). GO enrichment analysis of the 428 pleiotropic genes revealed significant overrepresentation in several key biological processes including modification of synaptic structure (*p* = 7.94 × 10^−5^), regulation of protein catabolism (*p* = 8.36 × 10^−5^), substantia nigra development (*p* = 0.0001), ethanol catabolism (*p* = 0.0002), and neuronal apoptosis (*p* = 0.0003) (Supplementary Table 5). The KEGG pathway analysis demonstrated significant enrichment for genes involved in neurotrophin signaling pathway (*p* = 7.64 × 10^−5^), pathways of neurodegeneration-multiple diseases (*p* = 0.0001), spinocerebellar ataxia (*p* = 0.0003), amyotrophic lateral sclerosis (*p* = 0.0004), endometrial cancer (*p* = 0.0006), alzheimer disease (*p* = 0.002), huntington disease (*p* = 0.003), colorectal cancer (*p* = 0.003), FoxO signaling pathway (*p* = 0.0046) (Figure 3B).

### Causal effect of sarcopenia on cognitive impairment

**Figure 4:**
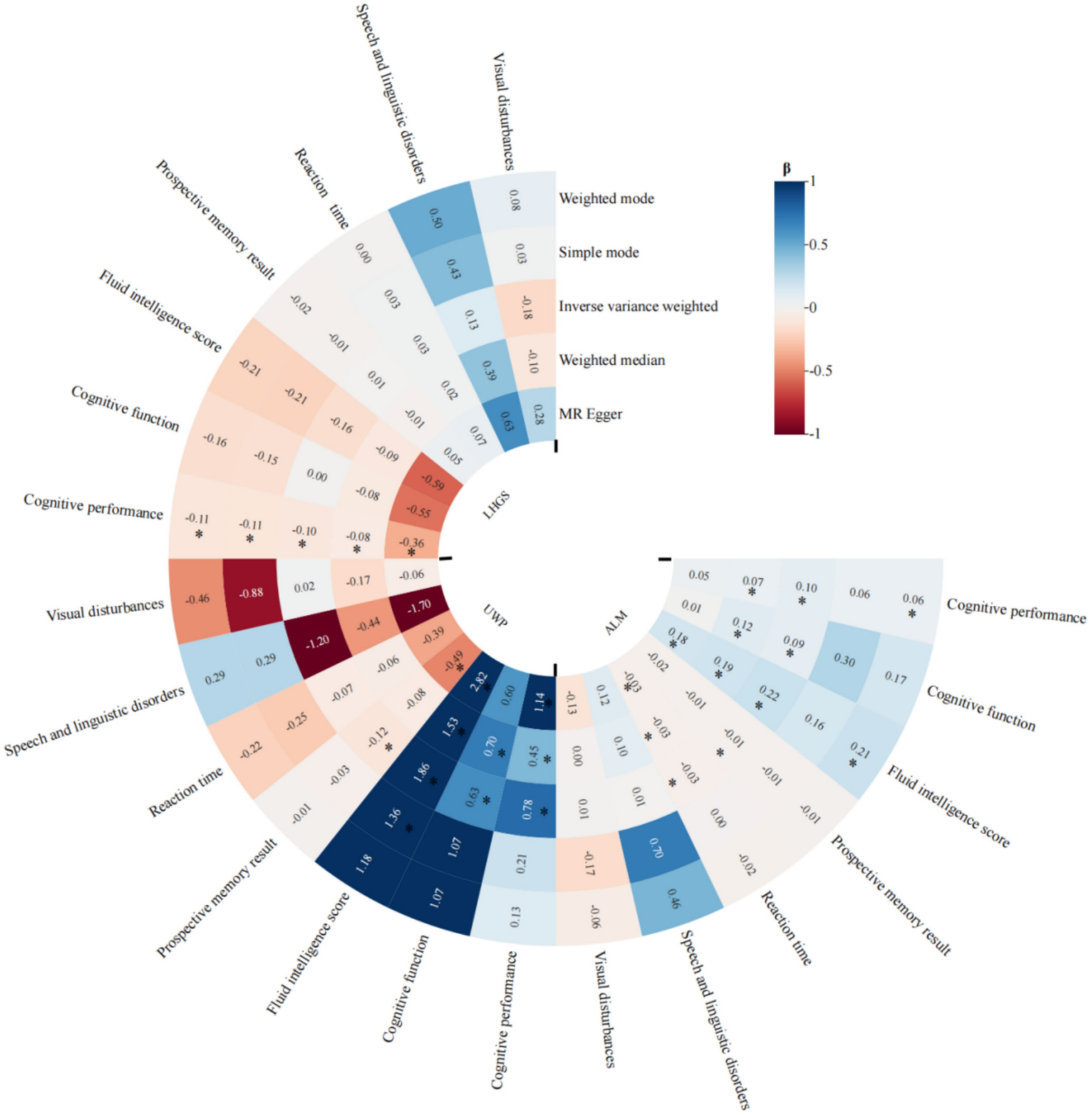
MR results of the causal effect of sarcopenia-related traits on 7 cognitive impairment outcomes. Date of ALM from ebi-a-GCST90000025, LHGS from ebi-a-GCST90007526, UWP from ukb-b-4711. And the cutoff level of LHGS was male < 30 kg, female < 20 kg by the European Working Group on Sarcopenia in Older People (EWGSOP). MR: mendelian randomization; ALM, appendicular lean mass; LHGS, low hand grip strength; UWP, usual walking pace; EWGSOP, European Working Group on Sarcopenia in Older People; β: beta values; * means *p* < 0.05.

**Figure 5:**
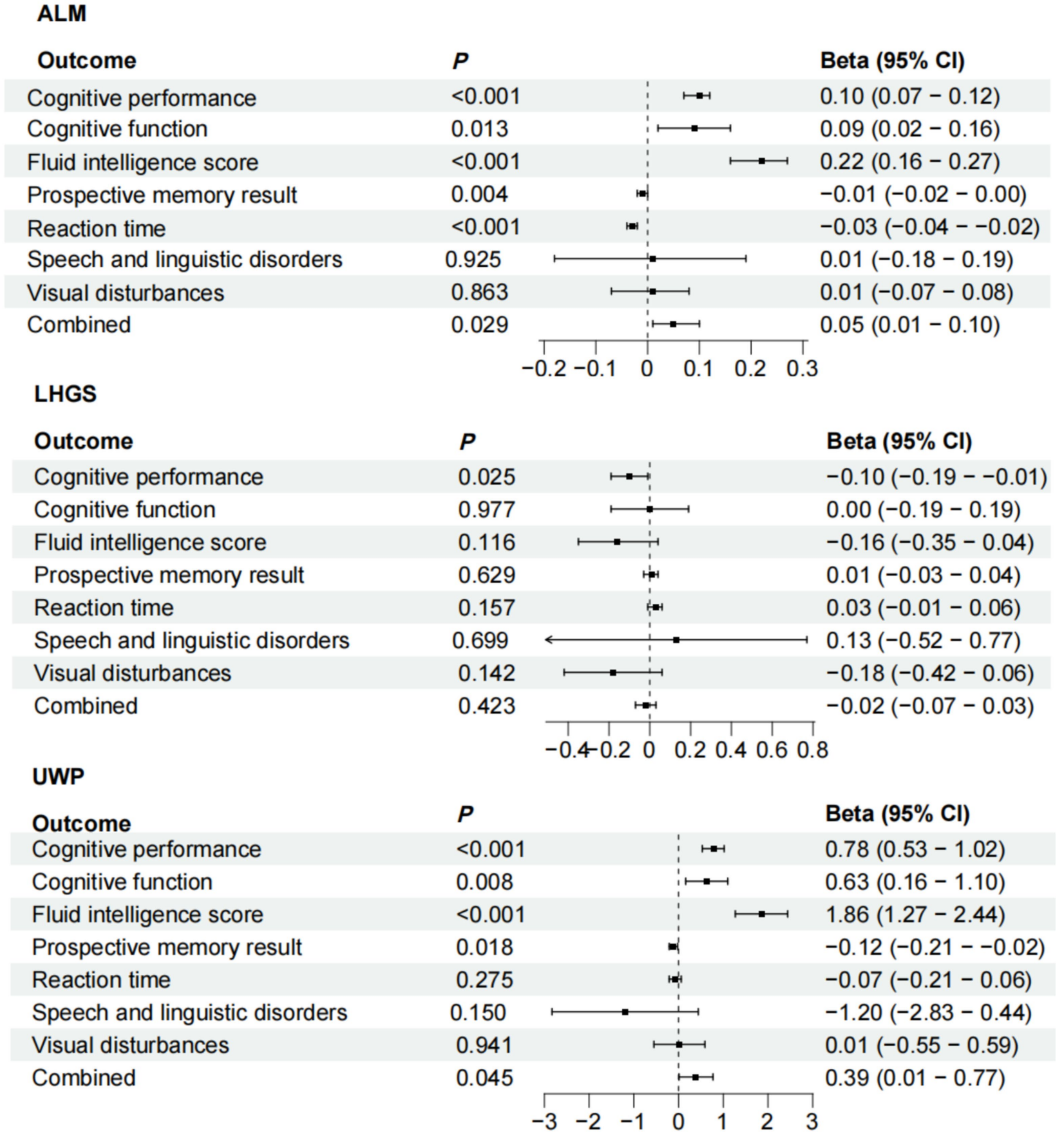
Meta-analysis of the causal associations between sarcopenia-related traits and 7 cognitive impairment outcomes. Date of ALM from ebi-a-GCST90000025, UWP from ukb-b-4711, LHGS from ebi-a-GCST90007526. And the cutoff level of LHGS was male < 30 kg, female < 20 kg by the EWGSOP. ALM, appendicular lean mass; LHGS, low hand grip strength; UWP, usual walking pace; EWGSOP, European Working Group on Sarcopenia in Older People.

To strengthen our causal inference, we conducted comprehensive MR analyses examining the directional relationships between sarcopenia-related traits and cognitive domains. The IVW method revealed statistically significant causal associations of appendicular lean mass (ALM) with multiple cognitive measures including cognitive performance (*p* < 0.001), improved global cognitive function (*p* = 0.013), fluid intelligence scores (*p* < 0.001), reaction time (*p* = 0.004), and prospective memory performance (*p* < 0.001). Our analysis revealed that LHGS exhibited a causal link exclusively with cognitive performance (*p* = 0.025), with no significant associations detected for other cognitive impairment related traits. We also found potential causal relationship between the usual walking pace with cognitive performance (*p* < 0.001), cognitive function (*p* = 0.008), fluid intelligence score (*p* < 0.001), and reaction time (*p* = 0.018) (Figure 4). The meta-analyzed effect sizes demonstrated strong concordance with the primary MR findings across all examined traits, with the exception of LHGS which showed divergent results (Figure 5). We obtained similar results in the replicative cohort (Supplementary Figures 5, 6). All sensitivity results of MR are summarised in Supplementary Figures 7–9.

## Discussion

Clinical research results indicate that sarcopenia is significantly associated with cognitive impairment. Through an integrative multi-method genomic approach, we comprehensively examined both the shared genetic basis and putative causal associations between sarcopenia and cognitive impairment leveraging large-scale GWAS datasets. Our findings provide robust evidence for significant genetic correlations, pleiotropic mechanisms and causal relationships underlying these two conditions, shedding light on their interconnected biological pathways and potential clinical implications.

The present study demonstrates a robust association between sarcopenia and cognitive impairment in community-dwelling older adults. This association remained significant when examining individual components of sarcopenia (low grip strength and low muscle mass), reinforcing the link between muscle health and cognitive function. These results align with existing biological evidence suggesting shared pathways between muscle degeneration and cognitive decline, including chronic inflammation, metabolic dysregulation, and reduced neurotrophic support (Sui et al., 2020).

Through LDSC and HDL analysis, we found that there were 19 pairs of traits with significant genetic correlation between sarcopenia related phenotypes and cognitive impairment related traits. ALM and UWP were positively correlated with cognitive function, cognitive performance, and fluid intelligence, but negatively correlated with reaction time and prospective memory, suggesting that muscle mass and motor ability may have a protective effect on cognition. Conversely, our analyses revealed an inverse association between LHGS and cognitive function, while demonstrating positive associations with prolonged reaction time and increased risk of language disorders. This difference may reflect the difference in neuromuscular regulation mechanism between grip strength and other muscle phenotypes. In addition, the negative correlation of UWP with language disorders and visual abnormalities further supports the association between motor function and specific cognitive domains such as language and visual processing (Camargo et al., 2016). These results are consistent with epidemiological studies, that is, the decline of physical activity including slow walking speed is often accompanied by cognitive impairment (Wang et al., 2022), and the change of grip strength may more reflect the neurodegenerative process such as motor neuron or cerebellar dysfunction (Fellows et al., 2001).

By PLACO and MAGMA analysis, we identified 79 pleiotropic loci and 428 pleiotropic genes, among which FoxO3, SLC39A8 and RP11-436D23.1 were repeated in multiple trait pairs. FoxO3 is a key gene in the regulation of aging and oxidative stress (Cao et al., 2023). Previous studies have confirmed its role in muscle atrophy and neurodegenerative diseases (Aucello et al., 2009). SLC39A8 is involved in zinc ion transport. Abnormal zinc metabolism is associated with cognitive impairment and sarcopenia (Nebert and Liu, 2019). The enrichment analysis of these genes further revealed the common biological processes such as synaptic structure regulation, neuronal apoptosis and substantia nigra development, suggesting that neural plasticity and neuroprotective mechanisms may be the core link between sarcopenia and cognitive impairment (Ceccarelli and Solerte, 2025).

KEGG pathway analysis showed that pleiotropic genes were significantly enriched in neurotrophic factor signaling pathway, neurodegenerative disease pathways including Alzheimer’s disease, Huntington’s disease and FoxO signaling pathway. FoxO pathway plays an important role in muscle protein metabolism and neuron survival, which may affect muscle and brain function by regulating autophagy, inflammatory response and other mechanisms (Penniman et al., 2023; Asadi et al., 2025). In addition, the enrichment of genes related to ethanol metabolism suggests that lifestyle factors such as alcohol intake may aggravate the risk of comorbidity through epigenetic modification (Fernandez-Solà et al., 2007; Peng et al., 2020).

MR analysis showed that ALM and UWP had potential causal protective effects on cognitive impairment related traits including cognitive function, fluid intelligence, while LHGS was only related to cognitive performance. This result supports that muscle mass rather than pure grip strength has an independent impact on cognitive health, which may be related to muscle factors secreted by muscle such as irisin promoting neurogenesis through the blood–brain barrier (Wang et al., 2025). The causal effect of UWP is particularly significant because it comprehensively reflects muscle function, cardiopulmonary endurance and nervous system integration ability (Lin et al., 2024; Zukowski et al., 2024).

However, colocation analysis found no shared causal genetic loci, suggesting that the association between sarcopenia and cognitive impairment may be mediated by pleiotropic genes rather than direct causal mutations. This finding suggests that our treatment for common pathways such as FoxO or neurotrophic factor signaling may improve muscle and cognitive function at the same time, but simply improving grip strength may not delay cognitive decline (Goh et al., 2024; Sajan et al., 2016; Yang et al., 2025).

This study has the following limitations: firstly, the data are mainly based on the European population and need to be verified in other ethnic groups. Secondly, the functional mechanism of pleiotropic genes such as non-coding RNA RP11-436D23.1 is not clear. Thirdly, MR analysis cannot completely exclude potential confounding factors such as vascular risk or chronic inflammation. Finally, we did not explore whether diet quality, physical activity, and energy balance interact with genetic susceptibility affecting muscle and cognitive function.

## Conclusion

This study provides evidence for both clinical links between sarcopenia and cognitive impairment. In addition, this study confirmed the shared genetic basis of sarcopenia and cognitive impairment through genetic analysis, and pleiotropic genes and neurodegenerative pathways are the core mechanisms of their association. Appendicular lean mass and walking pace may have a causal protective effect on cognitive function, while the effect of grip strength is more complex. In-depth research on the combined effects of genetics and lifestyle on sarcopenia and cognitive impairment might offer new insights into the treatment for sarcopenia and cognitive impairment.

## Data Availability

Publicly available datasets were analyzed in this study. This data can be found at: ieu open gwas.
